# Supplementary material for: Exploring anxiety symptoms, coping strategies, and socio-demographic influences among myocardial infarction patients in a tertiary care setting
Source: PLoS One. 2025 May 21;20(5):e0323904. doi: 10.1371/journal.pone.0323904 (PMC12094772; doi:10.1371/journal.pone.0323904)
Supplement: Attachment — Submitted filename: Comments_s.docx [file pone.0323904.s001.docx]

Thank you for submitting a research manuscript on a highly relevant topic: patients with myocardial infarctions, their anxiety, and coping skills. However, there are significant revisions needed to enhance the quality of the manuscript. These revisions are outlined below.

It is important to note that commenting on this manuscript is challenging due to the absence of line numbers and the line spacing not adhering to PLOS One manuscript guidelines.

**Major revisions**

*1.Introduction :*

The authors should pay closer attention to properly citing the literature used throughout the manuscript as not citing sources properly is considered plagiarism. E.g.

- In the first paragraph - The first sentence (the source for the definition of anxiety) needs a citation
- The second and fourth sentences also need to be cited
- In the second para – The first sentence needs a citation
- In the third para – the second and third statements are these from the same article as [5]?
- In the 6^th^ para (before methods) etc.

In the presentation of the literature, it would be most effective to structure it in a manner that describes international evidence, followed by regional evidence, and then concluding with local evidence. This organization will enhance clarity and understanding.

*2.Materials and Methods:*

Second para – second sentence – So, was the final questionnaire administered in English or Urdu?

Has the HAM tool or the COPE scale previously been validated in Pakistan? If not, the authors should at least describe the cultural adaptation process of the tools.

Third para- the last sentence is not clear- Is it the same participants or the same sample population? Using the same participants to pre-test the questionnaire will invariably introduce bias.

Who were the data collectors? Health staff? Using healthcare workers to conduct data collection, especially when it involves questions about the satisfaction of services, does not seem ideal as it will induce bias.

The authors have used non-probability convenient sampling to capture the sample size of 203 individuals. However, the whole analysis depended on the assumption that the data is normally distributed. It is important to note that data from non-probability samples may not fit the assumptions of parametric tests such as the t-test. Looking at the data, gives an impression that the data is skewed. As the author guidelines state that the “authors must describe any analysis carried out to confirm the data meets the assumptions of the analysis performed (e.g. linearity, co-linearity, normality of the distribution)” suggests to include how the authors assumed that the data was normally distributed.

*3. Results:*

It would be better if the authors could explain the basis of categorizing the age into three categories 18-40, 41-60 and 61-85. The usual practice is to divide into age groups with equal intervals.

The participants were divided into lower, middle and higher socio-economic status. It would be beneficial for non-local readers of the manuscript to understand what these categories represent. Is it based on income? Assets or Education levels?

Tables: In column headings, first column, better to keep only the frequency and percentage and the Mean and SD have already been described in the text. No need for repetition.

*Discussion*

It's always better to summarise the findings of your research first and then compare it with available research evidence.

On all three regression models, the R^2^ values are very low 9%-16%. Even though it doesn't mean that your model is bad or worthless it’s worth discussing what it means (E.g. Maybe your model does not include all variables that are associated with the outcome)

The authors have focused on analysed data in the discussion section. However, the limitations and advantages of the study also needs to be included.

*4. Conclusion*

The last sentence – mentions optimizing physical health, while nowhere in the article physical health has been discussed. Please revisit.

**Minor**

In the 5th para – The second sentence is quite long, making it difficult to follow. Could you clarify what you mean? It would be helpful if you could break it down into several shorter sentences.

Under the results, in the title please correct brief COPE and HAM-A scores

The terms anxiety and coping scores and HAM-A and Brief COPE scores are used interchangeably throughout the manuscript. Suggest to use a uniform format.

Recreation drug users in the sample are quite high (36%). Is it a normal finding in the hospital set-up or a unique finding? Worthwhile to explain this to the international audience of this manuscript.

Tables need to be self-explanatory. Therefore, the symbols, short forms need to be described as a footnote
