# Supplementary material for: Exploring anxiety symptoms, coping strategies, and socio-demographic influences among myocardial infarction patients in a tertiary care setting
Source: PLoS One. 2025 May 21;20(5):e0323904. doi: 10.1371/journal.pone.0323904 (PMC12094772; doi:10.1371/journal.pone.0323904)
Supplement: Attachment — Submitted filename: Response to Reviewers.docx [file pone.0323904.s004.docx]

Thank you for consideration of our manuscript, we have tried our effort best to improve the work according to the revisions requested by the journal. We have responded to the points mentioned by the reviewers under:

Line number and spacing has been added appropriately.

**Major Revisions:**
*1. Introduction:*

- We have added references to all mentioned instances as well as where we found were necessary and missing in the original document

- We have made an effort to acknowledge the sequencing in the paragraph and have structured in a way that shifts from broader to more local research, i.e. international, to any one specific country to Pakistan (country of study)


*2. Materials and Methods:*

- The final questionnaire was administered in Urdu, and we have now made this clear in the text as well as the reason why.

- The tools have been validated in Pakistan, appropriate references have been added.

- It is not the same population, the pre-test population was not included in the final sample, and this has been made clear now in the manuscript.

- The data collectors were authors not working at NICVD, not part of the health staff there. This point has been made clear now in the manuscript.

- Reason for assumption of normal distribution has now been elaborated in the manuscript.


*3. Results:*

- Ages were categorized to better reflect different stages of adult life, this has been further elaborated and explained now in the manuscript.

- The participants were divided based on income, explanation along with cut off values for different classes has been mentioned in the manuscript now.

- The tables have been heavily revised now, both to reduce redundancy and also to generally look neater and easier to interpret/read.

*Discussion*

- We have re-arranged the discussion in a way which elaborates our own findings before discussion on other literature<

- We have added discussion on the regression models.

- Limitations and advantages of the study have now been elaborated on.


*4. Conclusion:*

- The mentioning of physical health has been removed.


**Minor Revisions:**

- The mentioned sentence has been re-written to hopefully be more comprehensible.

- Corrected.

- Uniform format for HAM-A and brief COPE scores used.

- High incidence of recreational drug users has been elaborated on in results section now.

- Footnotes have been added.
