# Supplementary material for: Exploring anxiety symptoms, coping strategies, and socio-demographic influences among myocardial infarction patients in a tertiary care setting
Source: PLoS One. 2025 May 21;20(5):e0323904. doi: 10.1371/journal.pone.0323904 (PMC12094772; doi:10.1371/journal.pone.0323904)
Supplement: Attachment — Submitted filename: Comments _s.docx [file pone.0323904.s003.docx]

The authors have significantly improved the manuscript with the recent amendments. However, a few minor issues still require attention and need to be addressed

Abstract

Line 48 – can re word to be more clear ‘e.g A cross-sectional study was conducted at the National Institute of Cardiovascular Disease (NICVD), Karachi, Pakistan, involving 203 patients diagnosed with myocardial infarction.

64-65 – suggest to remove physical health outcomes as it is not addressed in the current study

Introduction

Line 95 - The authors should clarify what the abbreviation HADS stands for.

Line 126 similar AKUADS

Line 143 to 148 – This sentence is very long, which makes its meaning confusing. Suggest to re word it

Line 148-152 Furthermore….. –It is unclear what point the authors are trying to make. Is it because it is a referral hospital or a specialized facility where patients from all over Pakistan visit?

Materials and Methods

164- Vicinity? The patients are from the NICVD. Vicinity gives the impression that the patients are from the surrounding area of the institute is it so?

171- To make the statement clear suggest adding ‘The questionnaire was first developed in English, and was translated into urdu….

192 – inclusion criteria

193 – where was the sample taken? different hospital

Results

239- it is customary to place the currency symbol at the beginning of the number, before the numerical value

In the tables, it is recommended to adopt a uniform format for decimal points. For example, in the first table authors use two decimal points, the second table uses one, and the 4^th^ and 5^th^ table alternates between two and three decimal points.

Table 8 – positive framing, last cell – alignment

References

Please pay more attention to references. There are a few errors. Include DOI/ Accessed date in appropriately. Suggest to recheck

e.g 625-627- reference 19 – not complete

re check references- 23, 24
