# Supplementary material for: Exploring anxiety symptoms, coping strategies, and socio-demographic influences among myocardial infarction patients in a tertiary care setting
Source: PLoS One. 2025 May 21;20(5):e0323904. doi: 10.1371/journal.pone.0323904 (PMC12094772; doi:10.1371/journal.pone.0323904)
Supplement: Attachment — Submitted filename: Response_to_Reviewers_auresp_2.docx [file pone.0323904.s005.docx]

| **Comments from Reviewers** | **Author’s responses** |
| --- | --- |
| Abstract  Line 48 – can re word to be more clear ‘e.g A cross-sectional study was conducted at the National Institute of Cardiovascular Disease (NICVD), Karachi, Pakistan, involving 203 patients diagnosed with myocardial infarction.  64-65 – suggest to remove physical health outcomes as it is not addressed in the current study | Thank you for your comments, your commitment to the betterment of the article is much appreciated!  - The sentence in question has now been reworded accordingly.  - The word in question has now been removed. |
| Introduction  Line 95 - The authors should clarify what the abbreviation HADS stands for.  Line 126 similar AKUADS  Line 143 to 148 – This sentence is very long, which makes its meaning confusing. Suggest to re word it  Line 148-152 Furthermore….. –It is unclear what point the authors are trying to make. Is it because it is a referral hospital or a specialized facility where patients from all over Pakistan visit? | Thank you for your comments, your commitment to the betterment of the article is much appreciated!  - Full form for both scales mentioned has now been included.  - The sentence in question has now been reworded to make it more comprehensible.   - The sentence in question was meant to emphasize how the study could be used as a reference to help map psychological states in myocardial infarction patients. Along with this it mentions how such info is potentially important for the prognosis of these patients. We realize mentioning the institution led to unnecessary confusion in the sentence and has subsequently been removed. |
| Materials and Methods  164- Vicinity? The patients are from the NICVD. Vicinity gives the impression that the patients are from the surrounding area of the institute is it so?  171- To make the statement clear suggest adding ‘The questionnaire was first developed in English, and was translated into Urdu….  192 – inclusion criteria  193 – where was the sample taken? different hospital | Thank you for your comments, your commitment to the betterment of the article is much appreciated!  - The word in question has been removed to avoid confusion.   - The sentence in question has been updated accordingly.  - Inclusion criteria is mentioned in line 208.  - Clarity has been provided. The pilot was conducted among patients in the same institute however these patients were **NOT** included in the final sample. |
| Results  239- it is customary to place the currency symbol at the beginning of the number, before the numerical value  In the tables, it is recommended to adopt a uniform format for decimal points. For example, in the first table authorsuse two decimal points, the second table uses one, and the 4^th^ and 5^th^ table alternates between two and three decimal points.  Table 8 – positive framing, last cell – alignment | Thank you for your comments, your commitment to the betterment of the article is much appreciated!  - Currency has now been mentioned before the number.  - We understand your recommendations. Although we have now adapted the 2^nd^ table to have two decimal points. We feel the 3 decimal points (in 4^th^, 5^th^ tables etc.) should remain the same, as changing this would have an effect on the accuracy of the results. There are several 3 decimal numbers in which the 2^nd^ decimal point is “0” altering this into 2 decimals could make the results look widely different especially for variables such as p value. We also feel if we selectively alter the ones that don’t have 0 in their second decimal point it would appear untidy.    - Cell has now been aligned accordingly. |
| References  Please pay more attention to references. There are a few errors. Include DOI/ Accessed date in appropriately. Suggest to recheck  e.g 625-627- reference 19 – not complete  re check references- 23, 24 | Thank you for your comments, your commitment to the betterment of the article is much appreciated!  - DOI has been added where available. Where it was not available for any article, a direct link has been added instead.  - The mentioned reference has now been fixed.  - References have been revised. |
