# Supplementary material for: Exploring anxiety symptoms, coping strategies, and socio-demographic influences among myocardial infarction patients in a tertiary care setting
Source: PLoS One. 2025 May 21;20(5):e0323904. doi: 10.1371/journal.pone.0323904 (PMC12094772; doi:10.1371/journal.pone.0323904)
Supplement: Attachment — Submitted filename: Response_to_Reviewers_auresp_3.docx [file pone.0323904.s006.docx]

All the authors of this study would like to thank the reviewers for their input and time invested in providing advice to bring the study to its optimum quality. We have addressed the recently mentioned comments from the reviewers below:

| Reviewer’s Comments | Author’s response |
| --- | --- |
| 1. 92 -treatment concerns, lack of support etc [4]. Studies in the past have highlighted the 93 prevalence of anxiety in the hospital setting-Please mention whether these patients were admitted with a life-threatening condition or for any other reason. | 1. Thank you for your comments! We have now elaborated more on the setting; i.e. newly admitted acute care patients. |
| 2. 115- significantly higher risks for anxiety (HR = 5.06) and depression (HR = 7.23 ) Please mention HR is refers to Hazard Ratios (HR) | 2. Thank you for your comments! We have now included in the full form of HR (i.e. Hazard ratio). |
| 3. 188 The questionnaire was given to the data collectors (authors of the study who did not 189 currently work at NICVD)-Could you clarify whether you're distributing the questionnaire within a specific time after admission in the study, or is it the time period given for participants to complete it | 3. Thank you for your comments! We have now elaborated further on when the questionnaire was distributed (i.e. on recruitment). |
| 4. 221- while 69 (34.0%) were female participants. Average age of the sample population was 54- hope this is mean ,if so please mention | 4. Thank you for your comments! Yes it was Mean, and we have now corrected it. |
| 5. 547 In conclusion, this study details the significant prevalence of anxiety among myocardial infarction patients and highlights the critical role of coping strategies in their psychological well-being. How do you say this it is not mention in the results ?? it should be significant associated factors among 203 patients | 5. Thank you for your comments! We have reworded the aforementioned sentence so that it is more accurate to our study. |
